# Prevalence of Hyperuricemia and Associated Cardiovascular Risk Factors in Elite Athletes Practicing Different Sporting Disciplines: A Cross-Sectional Study

**DOI:** 10.3390/jcm13020560

**Published:** 2024-01-18

**Authors:** Giuseppe Di Gioia, Simone Pasquale Crispino, Viviana Maestrini, Sara Monosilio, Maria Rosaria Squeo, Erika Lemme, Andrea Segreti, Andrea Serdoz, Roberto Fiore, Domenico Zampaglione, Antonio Pelliccia

**Affiliations:** 1 Institute of Sports Medicine and Science, National Italian Olympic Committee, Largo Piero Gabrielli 1, 00197 Rome, Italy; vivianamaestrini@gmail.com (V.M.); sara.monosilio@gmail.com (S.M.); marysqueo@yahoo.it (M.R.S.); erikalemme@msn.com (E.L.); andreaserdoz@gmail.com (A.S.); roberto.fiore@mail.com (R.F.); domzamp1@gmail.com (D.Z.); ant.pelliccia@gmail.com (A.P.); 2 Department of Cardiovascular Sciences, Fondazione Policlinico Universitario Campus Bio-Medico, Via Alvaro del Portillo 200, 00128 Rome, Italy; simone.crispino@unicampus.it (S.P.C.); a.segreti@policlinicocampus.it (A.S.); 3 Department of Movement, Human and Health Sciences, University of Rome “Foro Italico”, Piazza Lauro De Bosis 15, 00135 Rome, Italy; 4 Department of Clinical, Internal, Anesthesiologic and Cardiovascular Sciences, Sapienza University of Rome, Piazzale Aldo Moro 5, 00185 Rome, Italy

**Keywords:** athletes, hyperuricemia, metabolism, prevention, sport cardiology, cardiovascular risk

## Abstract

Uricemia has been identified as an independent risk factor for cardiovascular disease. In the general population, hyperuricemia is associated with hypertension, endothelial dysfunction, and other cardiovascular risk (CVR) factors. Our aim was to explore the prevalence of hyperuricemia among Olympic athletes, evaluating the influence of sporting discipline and its correlation with CVR factors. We enrolled 1173 Olympic athletes classified into four disciplines: power, skill, endurance, and mixed. Clinical, anthropometric data, and complete blood test results were collected. Hyperuricemia was present in 4.4% of athletes, 0.3% were hypertensive, 11.7% had high-normal blood pressure values, 0.2% were diabetic, 1.2%. glucose intolerance, 8.2% active smokers, and 3% were obese. Males had a higher prevalence of hyperuricemia (5.3%) than females (3.4%) with no significant differences between different sporting disciplines (male, *p* = 0.412; female *p* = 0.561). Males with fat mass >22% presented higher uricemia (5.8 ± 1 vs. 5.3 ± 1 mg/dL, *p* = 0.010) like hypertensive athletes (6.5 ± 0.3 vs. 5.3 ± 1 mg/dL, *p* = 0.031), those with high-normal blood pressure (5.13 ± 1 vs. 4.76 ± 1.1 mg/dL, *p* = 0.0004) and those with glucose intolerance (6 ± 0.8 vs. 5.3 ± 1 mg/dL, *p* = 0.066). The study provides a comprehensive evaluation of hyperuricemia among Olympic athletes, revealing a modest prevalence, lower than in the general population. However, aggregation of multiple CVR factors could synergistically elevate the risk profile, even in a population assumed to be at low risk. Therefore, uric acid levels should be monitored as part of the CVR assessment in athletes.

## 1. Introduction

Uricemia refers to the concentration of uric acid in the blood, a product of purine metabolism [1]. Hyperuricemia has been increasingly recognized as an independent risk factor for cardiovascular diseases (CVD), as elevated uric acid levels have been associated with hypertension, endothelial dysfunction, and atherosclerosis. Serum uric acid levels vary based on age, sex, and other factors [1].

The prevalence of hyperuricemia and its association with cardiovascular risk factors is a complex and multifaceted issue, it is accounted to be around 20%, although geographical and ethnical variability [2,3] exists. A recent study evaluated the prevalence of hyperuricemia and its association with cardiovascular risk factors, subclinical target organ damage, and CVD. It found the prevalence of hyperuricemia was 16.3% among 6927 patients. Furthermore, the presence of hyperuricemia has been associated with conditions such as left ventricular hypertrophy, atrial fibrillation, heart failure, and renal damage [4].

In the context of exercise, particularly high-intensity activities, transient spikes in uric acid levels have been observed [5]. These acute increases are attributed to increased purine metabolism and muscle catabolism during intense physical exertion. While short-term increases in uric acid levels are generally not a cause for clinical concern, prolonged hyperuricemia can have significant implications for athletes. These include increased cardiovascular risk, impaired kidney function, and musculoskeletal problems.

Furthermore, hyperuricemia has been shown to impair endothelial function by decreasing nitric oxide levels, thereby exacerbating underlying vascular disorder [1]. Indeed, hyperuricemia has been suggested to represent an independent risk factor for major adverse cardiovascular events [6]. Therefore, monitoring and management of uric acid levels should be considered an integral part of CVD prevention and treatment strategies in the general population, including athletes.

Our study aimed to assess the prevalence of hyperuricemia among Olympic athletes, evaluating the influence of sporting discipline and the correlation with cardiovascular risk factors.

## 2. Materials and Methods

The Institute of Sports Medicine and Science in Rome, a division of the Italian Olympic Committee, is responsible for the medical evaluation of elite athletes participating in the Olympics. Our study included a cohort of 1173 Olympic athletes, assessed over a decade from the London 2012 Summer Games to the Beijing 2022 Winter Olympic Games.

Athletes with thyroid diseases taking chronic thyroid hormones (*n* = 28), adrenal glands adenoma (*n* = 1) beta-blockers (*n* = 5), or diuretics taken for other medical diseases (*n* = 2) were excluded from the study.

Athletes were classified into four distinct sports disciplines [7]: power, skill, endurance, and mixed. Power disciplines included, among others, weightlifting, Greco-Roman wrestling and judo. Skill disciplines included archery, equestrian sports, and golf, to name a few. Endurance disciplines included, among others, cycling, rowing, and long-distance running. Mixed disciplines concerned sports such as football, volleyball, and basketball.

Anthropometric data, including height and body weight, were collected for each athlete, and body mass index (BMI) was subsequently calculated. Body composition and percentages of fat mass were ascertained using bioelectrical impedance analysis (BIA 101 Quantum, Akern, Pontassieve, Italy) with a constant sinusoidal current at an intensity of 50 kHz and 400 ºA. Dietary habits and nutritional components were also documented: nutritional history of macronutrients (protein, carbohydrates and fats), micronutrients (vitamins and minerals), supplements, hydration levels, and timing of meals according to exercise training were recorded.

Cardiovascular (CV) risk factors were defined as:

- Family history of CVD, considered if there were fatal or non-fatal CV events or an established diagnosis of CVD in first-degree male relatives younger than 55 years or in female relatives younger than 65 years [8].
- Regular cigarette smoking is defined as consumption of at least one cigarette per day.
- Fat mass, whose normal values are set between 10% and 22% for males and between 20% and 32% for females [9].
- Hypertension, defined as systolic blood pressure (SBP) higher than 140 mmHg and/or diastolic blood pressure (DBP) higher than 90 mmHg or if the subject was undergoing antihypertensive treatment [9]. High-normal blood pressure, defined as SBP between 130 and 139 mmHg and/or DBP between 85 and 90 mmHg [7].
- Diabetes was diagnosed if fasting glucose levels were ≥126 mg/dL or if the subject was taking insulin or antidiabetic drugs; glucose intolerance (GI) has been defined as fasting glucose values between 100 and 126 mg/dL [10].

Blood samples were collected after a minimum 10 h fasting period and tested (Analyzer Roche Diagnostics Cobas C311, Monza, Italy) on the same day in a consistent laboratory environment from 2012 to 2022. A comprehensive biochemical profile was obtained, including parameters such as complete blood count, ferritin, transferrin, uric acid, creatinine, lipid profile, glucose, C-reactive protein (CRP), erythrocyte sedimentation rate (ESR), thyroid-stimulating hormone (TSH), total cholesterol (TC), low-density lipoprotein (LDL), high-density lipoprotein (HDL), triglycerides (TG), and vitamin D.

Detection of serum uric acid was determined by the photometric enzymatic method, while glucose determination was by colorimetric enzymatic method.

Hyperuricemia was identified using sex-specific serum urate concentrations, with thresholds set at 7.0 mg/dL for men and 6.0 mg/dL for women [11,12].

The study design of the present investigation was evaluated and approved by the Review Board of the Institute of Medicine and Sports Science (date of approval: 23 February 2023; IRB approval code: cni2302202a). All athletes included in this study were fully informed of the types and nature of the evaluation and signed the consent form, according to Italian Law and Institute policy. All clinical data assembled from the study population are maintained in an institutional database. The work described has been carried out in accordance with The Code of Ethics of the World Medical Association (Declaration of Helsinki).

### Statistical Analysis

Categorical variables were represented as frequencies and percentages and subjected to either Fisher’s exact test or Chi-square test, contingent upon their appropriateness. For continuous variables, normality was ascertained before their presentation as means accompanied by standard deviations. These variables were then analysed using the Student’s *t*-test for independent samples, provided they conformed to a normal distribution. Comparative analyses across diverse sporting disciplines employed the Dunn test and Pairwise comparison techniques. The tables delineated the aggregated *p*-values corresponding to four predefined categories: Power (P), Skill (S), Endurance (E), and Mixed (M). A pairwise test was subsequently conducted if the aggregated *p*-value fell below the 0.05 threshold, which was set as the criterion for statistical significance. All statistical computations were executed using STATA Statistics for Windows, SE version 17 (College Station, TX, USA: StataCorp LLC).

## 3. Results

We studied 1173 athletes (636, 54.2% male), with a mean age of 26.5 ± 5.2 years old, prevalently Caucasian (only 43, 3.7% were Afro-Caribbean) practicing different sporting disciplines: 352 (30%) power, 152 (13%) skills, 279 (23.8%) endurance, 390 (33.2%) mixed.

CV risk factors were identified: four (0.3%) athletes (three males) were hypertensive, two were diabetic (one male insulin-dependent), ninety-six athletes (8.2%) were active smokers, two hundred forty-five (20.9%) had a family history of CVD, and thirty-five (3%) had BMI > 30 kg/m^2^.

Globally, hyperuricemia was present in fifty-two (4.4%) athletes, divided according to sporting discipline into: eleven (21.1%) power, eight (15.4%) skills, fifteen (28.8%) endurance and eighteen (34.6%) mixed. No significant differences in hyperuricemia prevalence between sporting disciplines were found. There were no significant gender-related differences: 34/636 (5.3%) in males vs. 18/537 (3.4%) female athletes (*p* = 0.098), with absolute higher values of serum uric acid in males (5.3 ± 1 mg/dL vs. 4.2 ± 0.9 mg/dL compared to females, *p* < 0.0001).

The main differences between male athletes with hyperuricemia and the control group are listed in Table 1.

No differences in age (*p* = 0.487) or anthropometric parameters (BMI, *p* = 0.807; body weight, *p* = 0.156) were found. Also, laboratory test results did not show differences in iron metabolism, lipid profile, inflammatory markers, and metabolic indexes. Furthermore, the composition of the diet was similar between athletes with hyperuricemia and those with normal serum values in both sexes.

Male athletes with hyperuricemia had higher values of fat mass (14.3 ± 5.4% vs. 12 ± 5.1% compared to those with normal values, *p* = 0.010). The same comparison was performed for female athletes (Table 2), confirming the absence of significant differences between the two groups (also in % fat mass, *p* = 0.316), with evidence of higher HDL levels (*p* = 0.008) and ferritin (*p* = 0.0002).

**Figure 1:**
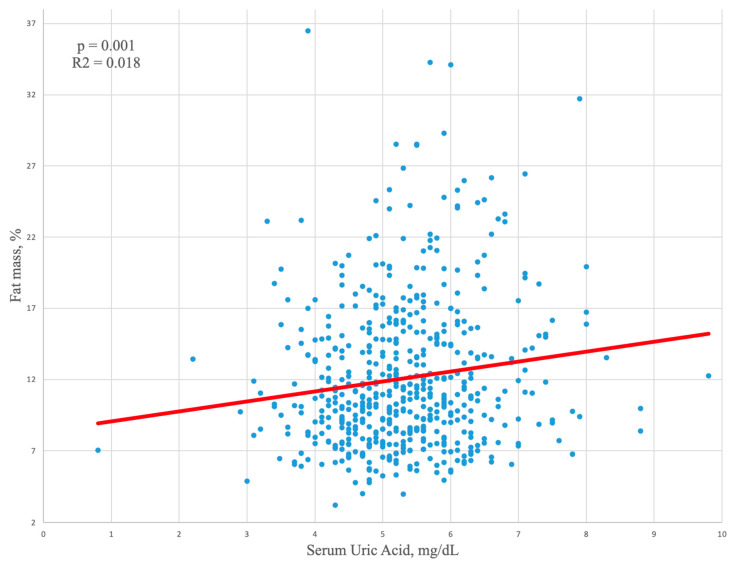
Linear regression analysis showing a positive correlation between fat mass and serum uric acid in male athletes.

In the relationship between uric acid and fat mass, gender differences were confirmed when we compared athletes with an excess of fat mass. In fact, in male athletes with a fat mass exceeding 22% (Table 3), higher uric acid values were found (5.8 ± 1 mg/dL vs. 5.3 ± 1 mg/dL in those with normal %, fat mass *p* = 0.010), while in females with fat mass > 32%, similar uric acid levels were found, compared to those with normal fat mass (*p* = 0.459). In male athletes, a positive linear correlation between fat mass and serum uric acid was found (Figure 1).

Furthermore, we evaluated serum uric acid according to the major CV risk factors (Table 3). In hypertensive male athletes (*n*= 3, 0.5%), mean uricemia was 6.5 ± 0.3 mg/dL, compared to 5.3 ± 1 mg/dL in normotensive athletes (*p* = 0.031). Also, males with GI (eight athletes, 1.2%) showed higher uric acid values (6 ± 0.8 mg/dL vs. 5.3 ± 1 mg/dL), close to statistical significance (*p* = 0.066). One male athlete had insulin-dependent diabetes with normal serum uric acid. A total of 137 athletes (11.7%) had high-normal blood pressure; of these, 124 (90.5%) were male. Also, in male athletes with a pre-hypertensive status, high values of glycemia and serum uric acid were found (Table 4). In males with SBP 130–139 mmHg and/or DBP 85–90 mmHg (*n* = 124, 10.6%), higher glycemia and uricemia values were found compared to normotensive athletes (respectively 95.7 ± 7.5 mg/dL vs. 91.4 ± 7 mg/dL, *p* < 0.0001 for glycemia and 5.13 ± 1 mg/dL vs. 4.76 ± 1.1 mg/dL, *p* = 0.0004 for uricemia). Similar results were found when athletes with isolated high-normal SBP or high-normal DBP were considered. In fact, in those with high-normal SBP, glycemia was 94.6 ± 7.5 mg/dL (*p* = 0.0003) and uricemia 5.12 ± 0.9 mg/dL (*p* = 0.004); while in those with isolated high-normal DBP, glycemia was 97.2 ± 7.2 mg/dL (*p* < 0.0001) and uricemia 5.24 ± 1 mg/dL (*p* = 0.014) compared to normotensive male athletes. Further, we evaluated changes of hyperuricemia over time and influence on possible development of CV risk factors: long-term clinical and blood tests data were available for 39/52 (75%) athletes with hyperuricemia, with a mean follow-up of 64.3 ± 31.9 months. In those athletes, at t0 serum uric acid was 6.9 ± 0.9 mg/dL with similar values at t1 (7 ± 1.1 mg/dL, *p* = 0.818) with most of athletes with hyperuricemia persisting with altered values at follow-up (*n* = 33, 84.6%). Similar mean values at follow-up were noted also for glycemia (93.1 ± 10.4 mg/dL at t0 vs. 92 ± 8.4 mg/dL at t1, *p* = 0.536). However, four (10.2%) new cases of GI were identified. No athletes developed hypertension at follow-up, but in three athletes (7.7%) high-normal values were highlighted. In fact, for both SBP and DBP higher mean values were found at follow-up, close to statistical significance for SBP (106.7 ± 10.2 mmHg at t0 vs. 111.4 ± 10.1 mmHg, *p* = 0.069 and 67.4 ± 7.8 mmHg at t0 vs. 69.3 ± 8.3 mmHg at t1, *p* = 0.335 for DBP).

Finally, we evaluated the influence of sporting discipline (Table 5). In both male and female athletes, similar serum uric acid concentrations were found in different sports disciplines: in males 5.2 ± 1 mg/dL in power disciplines, 5.5 ± 0.9 mg/dL in skills, 5.2 ± 1.1 mg/dL in endurance, and 5.4 ± 0.9 mg/dL in mixed sports (*p* = 0.412); in females 4.1 ± 0.8 mg/dL in power disciplines, 4.1 ± 0.8 mg/dL in skills, 4.3 ± 0.9. mg/dL in endurance and 4.2 ± 0.9 mg/dL in mixed sports (*p* = 0.516).

## 4. Discussion

The study presents a comprehensive examination of hyperuricemia and cardiovascular risk factors among a large cohort of Olympic athletes. The prevalence of hyperuricemia in our cohort is 4.4% and is lower compared to the general population, which is accounted to be around 20%, although there is geographical and ethnical variability [2,3].

The reported prevalence of hyperuricemia in athletes is variable. A study by Kuo et al. [5] explored the relationship between anthropometric indices and hyperuricemia among adolescent athletes in Northern Taiwan. The study involved 387 student-athletes (218 males and 169 females) with a mean age of 17.4 years. The prevalence of hyperuricemia was found to be 27.1% in male and 21.8% in female adolescent athletes. This prevalence was notably higher than that in general adolescents, approximately twice that reported in nationally representative U.S. adolescents (10.9%) [5]. These findings suggested hyperuricemia was relatively common among young athletes and comparable to prevalence rates in the general population, although different from our observations.

No clear evidence on elite athletes was available so far. In our cohort, we found lower prevalence when compared to the general population and to the previous studies. This may be attributed to the enhanced cardiovascular and metabolic urate excretion efficiency associated with regular physical activity that characterizes these elite individuals.

Actually, the direct impact of hyperuricemia on major adverse cardiovascular events in athletes is not well-established. While high uric acid levels are associated with an increased risk of cardiovascular events in the general population [1], the protective effects of regular physical activity in athletes may offset this risk, although no longitudinal data on athletes in this setting are available.

In effect, athletes typically exhibit a favourable cardiovascular risk profile, including lower rates of hypertension, obesity, and dyslipidaemia, compared to the general population [7]. This profile is largely due to regular physical activity, which improves lipid profiles, enhances insulin sensitivity, and lowers blood pressure [13].

Nevertheless, the relationship between hyperuricemia, other CVRFs, and major cardiac events (MACEs) is intricate. In fact, while this correlation is well acknowledged in the general population, in the athletic population more caution is needed. Elevated uric acid levels have been associated with an increased risk of myocardial infarction, stroke, and cardiovascular death [6]. A meta-analysis by Kim et al. [14] demonstrated hyperuricemia was associated with a higher risk of MACE, particularly in patients with pre-existing CVD [14].

Hypertension is one of the main cardiovascular risk factors and, in our cohort, we found a low prevalence of hypertension among athletes (0.3%), underlining the concept that regular physical activity in athletes tends to lower blood pressure. However, while aerobic exercise generally reduces BP, resistance exercise may both have a neutral or elevating effect on BP, for which periodical controls are advised [15]. In this setting, we found hypertensive athletes presented higher values of uricemia when compared to non-hypertensive. Several epidemiological studies have demonstrated a strong correlation between elevated uric acid levels and the development of hypertension. A study by Forman et al. [16] found higher uric acid levels were associated with an increased risk of incident hypertension, particularly in younger and leaner individuals.

In our study, we found, in most of the athletes, hyperuricemia tended to persist over time with a tendency to have higher values of both SBP and DBP at follow-up.

Moreover, even in athletes with high-normal blood pressure values, significantly higher serum concentrations of uric acid were found. In our population, it was not a rare condition, present in 11.7% of athletes, mainly highlighted in males, and it also was associated with higher fasting glycemia.

This may reflect the fact that elevated uric acid levels can induce endothelial dysfunction, reduce nitric oxide availability, and lead to vasoconstriction, thereby increasing blood pressure [1]. Additionally, uric acid can stimulate the renin-angiotensin system and reduce the levels of natriuretic peptides, further contributing to hypertension [17].

Among the CVRFs, in the general population, a study investigated the relationship between hyperuricemia and metabolic syndrome finding hyperuricemia is significantly correlated with hypertriglyceridemia, hypertension, and visceral obesity [18].

In this sense, our study highlights a relationship between uric acid levels and % fat mass in male athletes. This finding is supported by the literature demonstrating increased adiposity, particularly visceral fat, is associated with higher uric acid levels due to insulin resistance and increased purine turnover [5,18].

Actually, the interplay between hyperuricemia and other metabolic parameters such as triglycerides, glycemia, and body mass index (BMI) in athletes is complex.

Triglycerides have been linked to CV risk factors [3]. Elevated triglyceride levels may suggest an underlying metabolic syndrome, which is often accompanied by hyperuricemia [5,18]. In this sense, we may suppose the relationship between triglycerides and uric acid could be bidirectional, in fact, high levels of uric acid may contribute to dyslipidaemia and vice versa. Furthermore, a higher BMI often correlates with increased adipose tissue, which can lead to insulin resistance and, consequently, elevated uric acid levels [5]. In athletes, especially those in weight-sensitive sports, a higher BMI could be indicative of increased muscle mass rather than fat, yet the metabolic implications remained similar.

In effect, our study also showed the relationship between uric acid levels and fat mass. In male athletes with fat mass exceeding 22%, higher uric acid values were found. We suggested a potential link between uric acid levels and body composition, at least in male athletes since this relationship was not observed in female athletes, even those with a fat mass greater than 32%. Findings are confirmed by the aforementioned study of Kuo et al. [5] which additionally found obesity-related anthropometric parameters like increased BMI, waist circumference, and body fat percentage (BF%) were significantly associated with hyperuricemia in both genders. The study also found a U-shape association between BF% and the prevalence of hyperuricemia, indicating both high and low BF% are associated with elevated serum uric acid levels.

In the metabolic framework, glucose intolerance and insulin resistance may also emerge as determining factors. Glucose is known to cause intolerance, decrease insulin sensitivity, and potentially lead to hyperinsulinemia or diabetes. Hyperuricemia works by promoting the reabsorption of uric acid in the kidney tubules, which can increase the formation of fat cells in the liver [4]. This process further disrupts purine metabolism, resulting in a deeper increase in serum uric acid levels.

Of note, also insulin resistance causes an increase in uric acid synthesis via the hexose monophosphate pathway which reduces its renal excretion [19]. Furthermore, excess adipose tissue is a key indicator of insulin resistance, potentially contributing to oxidative stress. This relationship may show the complex interaction between insulin resistance, glucose intolerance, and hyperuricemia, whose interconnections highlights the importance of understanding and managing these factors in clinical settings, particularly in the management of metabolic disorders and cardiovascular risk.

In addition, we found several differences between the male and the female population. Prevalence resulted in higher, albeit not statistically significant, in males (5.3%) compared to females (3.4%). This gender difference was intriguing and may warrant future investigation, especially given males with hyperuricemia presented higher values of fat mass. First, males’ greater muscle mass could lead to increased purine turnover and uric acid production post-exercise, as described [20]. Additionally, higher circulating testosterone in males can enhance renal uric acid reabsorption, while estrogen in females may modulate purine metabolism and promote excretion [21]. Sex-specific genetic factors and hormonal regulation of blood pressure and vascular function may also play roles.

Eventually, while hyperuricemia could independently serve as a CV risk factor in an otherwise healthy athletic population, our data suggested other traditional CV risk factors were not absent among these elite athletes and had a close relationship.

In conclusion, although a modest 0.3% were hypertensive and a mere 0.2% were diabetic, actually 8.2% were active smokers, 3.9% had higher fat mass than normal, and 20.9% had a family history of CVD. Moreover, those with high blood pressure and GI presented higher values of serum uricemia compared to the control group.

Our findings may support the hypothesis that the aggregation of multiple and discrete risk factors could synergistically elevate the CV risk profile, even in a population generally assumed to be at low risk due to their elite athletic status. Hence, emphasis should be given to the management of uricemia levels in elite athletes, especially in those presenting additional non-modifiable risk factors.

## 5. Strengths and Limitations

The study presents several strengths that contribute to its validity. First, the sample size is robust and consists of 1173 Olympic athletes, an acknowledged difficult population to assemble. The inclusion of athletes from a wide range of sports disciplines improves the generalizability of the results.

However, the study is not free from limitations. The study’s cross-sectional nature may limit causal inference. The absence of a non-athlete control group may limit the broader interpretation of the findings, although considerations for the general population may not apply to athletes and vice versa. The limited ethnic diversity, mainly Caucasian (96.3%), may limit the applicability of the results to other demographic groups.

Serum uric acid concentrations could be influenced by training intensity and quantity: athletes enrolled in our study followed very different and heterogeneous training programs. However, our evaluation was performed in different training periods and even if most of the athletes did not exercise the day before the blood test sample collection, several athletes did not follow a rest period superior to 24 h, with possible influence of serum uric acid levels.

Finally, while the study found a correlation between elevated uric acid levels and hypertension, glucose intolerance, and fat mass among elite athletes, the small sample size (only three athletes with hypertension) may challenge the validity of this association. Consequently, asserting a direct link between elevated uric acid and increased long-term cardiovascular risk in these athletes may be premature based on the current data. Moreover, some results approached but did not reach statistical significance, indicating potential limits of statistical power for certain subanalyses.

## 6. Conclusions

The study underscores the importance of monitoring uric acid levels in athletes, not merely as a marker for gout but as a potential cardiovascular risk factor since the prevalence of hyperuricemia in athletes is lower than in the general population but is not so rare. In addition, the aggregation of multiple risk factors could synergistically elevate the cardiovascular risk profile, even in a population generally assumed to be at low risk due to their elite athletic status. Therefore, uric acid levels should be monitored as part of cardiovascular risk assessment in athletes.

## Figures and Tables

**Table 1:** General, anthropometrics, diet, sporting discipline and laboratory differences between male athletes with hyperuricemia and the control group.

MALE	N, 636	Hyperuricemia	Normal Uricemia	*p*
	N, (%)	34 (5.3)	602 (94.7)	-
General	Age, years	27.7 ± 5.8	27 ± 5.2	0.487
	Afro-Caribbean, *n* (%)	2 (5.9)	26 (4.3)	0.665
	Smokers, *n* (%)	4 (11.8)	51 (8.5)	0.430
	Familiarity for CVD, *n* (%)	7 (20.6)	130 (21.6)	0.889
Anthropometrics	Weight, kg	84.7 ± 13.3	81.3 ± 13.6	0.156
	BMI, kg/m^2^	25 ± 3.3	24.5 ± 3	0.807
	Fat mass, %	14.3 ± 5.4	12 ± 5.1	0.010
	Waist, cm	88.3 ± 10.7	90.2 ± 20.2	0.742
Diet	Kcal	2936.9 ± 822.5	2960.9 ± 692.3	0.876
	Protein, %	20 ± 4.8	19.5 ± 3.9	0.590
	Fat, %	30.1 ± 4.1	29.7 ± 3.3	0.594
	Carbohydrate, %	50.1 ± 7.1	50.7 ± 5.2	0.587
Sporting discipline	Power, *n* (%)	9 (26.5)	185 (30.7)	0.599
	Skills, *n* (%)	7 (20.6)	80 (13.3)	0.228
	Endurance, *n* (%)	9 (26.5)	160 (26.6)	0.989
	Mixed, *n* (%)	9 (26.5)	177 (29.4)	0.714
Lab Results	Uricemia, mg/dL	7.6 ± 0.6	5.2 ± 0.8	<0.0001
	ESR, mm/h	2.6 ± 1.5	2.7 ± 2.6	0.803
	CRP, mg/mL	1 ± 1.1	1 ± 2	0.966
	Hb, g/dL	14.5 ± 2.7	14.8 ± 1.9	0.412
	MCV, fL	86.6 ± 3	85.9 ± 10.8	0.746
	HCT, %	44.2 ± 2	43.2 ± 5.7	0.408
	PTL	214.5 ± 58.5	209.8 ± 45.1	0.614
	Ferritin, ng/mL	190 ± 125.2	183.5 ± 114.4	0.755
	Iron, mcg/dL	107 ± 44.3	103 ± 35.7	0.527
	Transferrin, mg/dL	247.2 ± 33.7	239.2 ± 28.7	0.120
	TSH	2.2 ± 1	2.4 ± 3.5	0.804
	Glycemia, mg/dL	93.6 ± 8.3	93.2 ± 6.9	0.778
	Vitamin D	39.3 ± 10.4	37.1 ± 12.2	0.581
	TC, mg/dL	175.9 ± 34.1	174.3 ± 32.1	0.778
	LDL, mg/dL	97 ± 28.3	99.2 ± 28.8	0.660
	HDL, mg/dL	62.5 ± 18.5	59.6 ± 13.9	0.254
	TG, mg/dL	85.7 ± 52.4	79.8 ± 48.6	0.493
	LDL/HDL	1.68 ± 0.7	1.75 ± 0.7	0.542

Abbreviations: BMI: body mass index; CRP: C-reactive protein; CVD: cardiovascular disease; ESR: erythrocyte sedimentation rate; Hb: hemoglobin; HCT: hematocrit; HDL: high-density lipoprotein; LDL: low-density lipoprotein; MCV: mean corpuscular volume; PTL: platelet; TC: total cholesterol; TG: triglycerides; TSH: thyroid stimulating hormone.

**Table 2:** General, anthropometrics, diet, sporting discipline and laboratory differences between female athletes with hyperuricemia and the control group.

FEMALE	N, 537	Hyperuricemia	Normal Uricemia	*p*
	N, (%)	18 (3.4)	519 (96.6)	-
General	Age, years	26.3 ± 4.3	25.8 ± 5	0.681
	Afro-Caribbean, *n* (%)	0 (0)	15 (2.9)	0.464
	Smokers, *n* (%)	2 (11.1)	39 (7.5)	0.572
	Familiarity for CVD, *n* (%)	4 (22.2)	104 (20)	0.823
Anthropometrics	Weight, kg	66.8 ± 1.7	63.6 ± 11.3	0.238
	BMI, kg/m^2^	22.5 ± 1.7	22 ± 3	0.453
	Fat mass, %	19.2 ± 5.7	20.6 ± 5.5	0.316
	Waist, cm	89.8 ± 20	81 ± 20.3	0.145
Diet	Kcal	2228.6 ± 489.1	2267.7 ± 4	0.844
	Protein, %	20.7 ± 3.9	19.2 ± 4	0.349
	Fat, %	27.7 ± 2	29.4 ± 4.2	0.290
	Carbohydrate, %	51.6 ± 3.1	50.8 ± 5.7	0.739
Sporting discipline	Power, *n* (%)	2 (11.1)	156 (30)	0.082
	Skills, *n* (%)	1 (5.6)	64 (12.3)	0.386
	Endurance, *n* (%)	6 (33.3)	104 (20)	0.169
	Mixed, *n* (%)	9 (50)	195 (37.6)	0.285
Lab Results	Uricemia, mg/dL	6.4 ± 0.4	4.1 ± 0.8	<0.0001
	ESR, mm/h	2.7 ± 1.2	4.9 ± 4.3	0.081
	CRP, mg/mL	0.7 ± 0.6	1.2 ± 2.5	0.344
	Hb, g/dL	13.6 ± 1	13.4 ± 1.7	0.650
	MCV, fL	87.3 ± 8.5	88.4 ± 8.4	0.601
	HCT, %	40.4 ± 2.5	39.7 ± 3.6	0.411
	PTL	230.6 ± 38.9	233.1 ± 50.7	0.848
	Ferritin, ng/mL	116.3 ± 94.1	68.8 ± 51.5	0.0002
	Iron, mcg/dL	108.5 ± 51	101.9 ± 41.7	0.507
	Transferrin, mg/dL	267.6 ± 40.7	261 ± 42.2	0.513
	TSH	2.3 ± 1.1	2.2 ± 1.1	0.767
	Glycemia, mg/dL	89.4 ± 7	89.9 ± 6.7	0.765
	Vitamin D	31.1 ± 6.8	38.4 ± 16	0.235
	TC, mg/dL	187.4 ± 29.3	177.8 ± 32.6	0.219
	LDL, mg/dL	90.8 ± 21.3	93.8 ± 25	0.609
	HDL, mg/dL	80.9 ± 18.5	71.4 ± 14.8	0.008
	TG, mg/dL	79.6 ± 49.6	67.9 ± 26.4	0.077
	LDL/HDL	1.17 ± 0.4	1.46 ± 2.2	0.588

Abbreviations: BMI: body mass index; CRP: C-reactive protein; CVD: cardiovascular disease; ESR: erythrocyte sedimentation rate; Hb: hemoglobin; HCT: hematocrit; HDL: high-density lipoprotein; LDL: low-density lipoprotein; MCV: mean corpuscular volume; PTL: platelet; TC: total cholesterol; TG: triglycerides; TSH: thyroid stimulating hormone.

**Table 3:** Differences in serum uric acid in male athletes according to an excess of fat mass, hypertension, and glucose intolerance.

**MALE; N = 636**	**Fat mass ≤ 22%**	**Fat mass > 22%**	***p***
N, (%)	606 (95.3)	30 (4.7)	-
Uricemia, mg/dL	5.3 ± 1	5.8 ± 1	0.010
**FEMALE; N = 537**	**Fat mass ≤ 32%**	**Fat mass > 32%**	***p***
N, (%)	521 (97)	16 (3)	-
Uricemia, mg/dL	4.2 ± 0.9	4.4 ± 0.8	0.459
**MALE; N = 636**	**HBP**	**Control group**	***p***
N, (%)	3 (0.5)	633 (99.5)	-
Uricemia, mg/dL	6.5 ± 0.3	5.3 ± 1	0.031
**MALE; N = 636**	**GI**	**Control group**	***p***
N, (%)	8 (1.2)	628 (98.8)	-
Uricemia, mg/dL	6 ± 0.8	5.3 ± 1	0.066

Abbreviations: GI: glucose intolerance; HBP: high blood pressure.

**Table 4:** Differences in serum uric acid levels and fast glycemia according to high-normal values of blood pressure in male athletes.

Male Athletes N = 636	Normotensive Athletes (BP < 130\85 mmHg)	High-Normal BP (SBP 130–139 mmHg AND\OR DPB 85–90 mmHg)	*p*	High-Normal SBP (130–139 mmHg)	*p*	High-Normal DBP (85–89 mmHg)	*p*
N, (%)	509 (43.4)	124 (10.6)		77 (6.6)		32 (2.7)	
Glycemia, mg/dL	91.4 ± 7	95.7 ± 7.5	<0.0001	94.6 ± 7.5	0.0003	97.2 ± 7.2	<0.0001
Uricemia, mg/dL	4.76 ± 1.1	5.13 ± 1	0.0004	5.12 ± 0.9	0.004	5.24 ± 1	0.014

Abbreviations: BP: blood pressure; DBP: diastolic blood pressure; SBP: systolic blood pressure.

**Table 5:** Serum acid uric variations in different sporting disciplines according to gender.

	Power	Skills	Endurance	Mixed	*p*
**Male athletes,** ***n*** **(%)**	194 (30.5)	87 (13.7)	169 (26.6)	186 (29.2)	-
Uricemia, mg/dL	5.2 ± 1	5.5 ± 0.9	5.2 ± 1.1	5.4 ± 0.9	0.412
**Female athletes,** ***n*** **(%)**	158 (29.4)	65 (12.1)	110 (20.5)	204 (38)	-
Uricemia, mg/dL	4.1 ± 0.8	4.1 ± 0.8	4.3 ± 0.9	4.2 ± 0.9	0.561

## Data Availability

De-identified participant data are available upon reasonable request from the corresponding author.
